# Biophysico-Chemical Properties of Alginate Oligomers Obtained by Acid and Oxidation Depolymerization

**DOI:** 10.3390/polym13142258

**Published:** 2021-07-09

**Authors:** Anna Zimoch-Korzycka, Dominika Kulig, Żaneta Król-Kilińska, Barbara Żarowska, Łukasz Bobak, Andrzej Jarmoluk

**Affiliations:** 1 Department of Functional Food Products Development, The Faculty of Biotechnology and Food Science, Wroclaw University of Environmental and Life Sciences, Chelmonskiego 37, 51-630 Wroclaw, Poland; anna.zimoch-korzycka@upwr.edu.pl (A.Z.-K.); zaneta.krol@upwr.edu.pl (Ż.K.-K.); lukasz.bobak@upwr.edu.pl (Ł.B.); andrzej.jarmoluk@upwr.edu.pl (A.J.); 2 Department of Biotechnology and Food Microbiology, The Faculty of Biotechnology and Food Science, Wroclaw University of Environmental and Life Sciences, Chelmonskiego 37, 51-630 Wroclaw, Poland; barbara.zarowska@upwr.edu.pl

**Keywords:** alginate, oligosaccharides, depolymerization, antioxidant properties, texture profile analysis

## Abstract

The aim of the study was to obtain alginate oligosaccharides by using two degradation methods of sodium alginate (SA): with hydrochloric acid (G—guluronate, M—mannuronate and G + M fractions) and hydrogen peroxide (HAS—hydrolyzed SA), in order to assess and compare their biological activity and physico-chemical properties, with an attempt to produce gels from the obtained hydrolysates. The efficiency of each method was determined in order to select the fastest and most efficient process. The ferric ion reducing antioxidant power (FRAP), the ability to scavenge DPPH free radicals, rheological properties, Fourier Transformed Spectroscopy (FTIR) and the microbiological test against *Escherichia coli* and *Staphylococcus aureus* were performed. In order to check the functional properties of the obtained oligosaccharides, the texture profile analysis was assessed. The hydrolysis yield of acid SA depolymerization was 28.1% and from hydrogen peroxide SA, depolymerization was 87%. The FTIR analysis confirmed the degradation process by both tested methods in the fingerprint region. The highest ferric reducing antioxidant power was noted for HSA (34.7 µg), and the highest hydroxyl radical scavenging activity was obtained by G fraction (346 µg/Trolox ml). The complete growth inhibition (OD = 0) of alginate hydrolysates was 1%. All tested samples presented pseudoplastic behavior, only HSA presented the ability to form gel.

## 1. Introduction

Polysaccharides are frequently subjected to certain modifications in order to enhance their capability to be applied in medical, engineering or food technologies. There are several methods to modify them, such as oxidation, reduction, esterification, cross-linking or hydrolysis, whereby the following compounds with the desired properties may be obtained. However, chemical modification of the polysaccharides is not specific, in contrast to enzymatic modification, which is characterized by high specificity of action [1]. 

The worldwide interest in oligosaccharides has increased since they were classified as prebiotics. Indigestible carbohydrates, in contrast to digestible carbohydrates, are not digested in the gastrointestinal tract. These include certain oligosaccharides and polysaccharides [2]. The effect of undigested carbohydrates may be present in three stages: (1) they may become intact to a large intestine, (2) selectively stimulate the intestinal microflora, and (3) exert beneficial effects on the body. Such carbohydrates are referred to as prebiotics and have a number of beneficial properties for the human body. Oligosaccharides, as prebiotics, may be functional food ingredients, reduce the intestinal pH, inhibit the growth of pathogenic microorganisms, improve the lipoprotein profile, facilitate the absorption of minerals, etc. [3].

Oligosaccharides are compounds of low molecular weight (*M_W_*) composed of 2–10 monosaccharide units. They may naturally occur in foods or may be prepared by the synthesis of disaccharides or by the hydrolysis of polysaccharides. Oligosaccharides can be obtained from fungi, bacteria, algae and higher plants. Oligosaccharides’ properties vary depending on the monosaccharide composition, molecular weight and chemical properties. These characteristics are directly related to the natural source from which they are extracted, as well as the presence of contaminants. They may be obtained by various methods, but those naturally available in the raw material need to undergo an extraction process. This can be carried out by dissolving the substrate in methanol or another alcohol solution and water. They can also be obtained by synthesis, which requires specific enzymes and controlled conditions. The synthesis process is expensive, difficult to reproduce on a larger scale and provides low yield [2]. The oligosaccharides can also be obtained by the depolymerization of polysaccharides. The production of oligosaccharides by this method is simpler, less expensive, and reproducible on an industrial scale. This treatment is most often carried out in order to change the physical properties of alginate, primarily a decrease in *M_W_*, which significantly improves its functional properties. Changing the three-dimensional structure of alginate, which affects its mechanical strength, demonstrated physiological and rheological properties, e.g., stability, viscosity, solubility in solutions and hydrophobicity, and can also lead to the improvement of the reactive properties of the compound [4,5]. Generally, sodium alginate is used in the food and beverage industry as a stabilizer, thickener in the preparation of drinks, ice cream, and jelly, as well as an encapsulation material of yeast cells in ethanol production. Unfortunately, sodium alginate does not possess biological activity which could expand its functional applications [5]. Sodium alginate oligosaccharides formed during the depolymerization process generally do not have the ability to form gels; however, they are characterized by other important biological activities. They affect the growth and rapid development of human cells, including epidermal cells, and prevent the occurrence of oxidative stress. They the promote growth of plants such as rice, lettuce, wheat, and tobacco. Sodium alginate oligomers have antimicrobial properties, are destructive to multi-drug-resistant bacteria, and are therefore used in the production of antibiotics. They contribute to the disturbed fungal growth of *Aspergillus* and *Candida* [6]. The health benefits of alginate oligosaccharides, such as immunomodulatory, antitumor, antioxidant, prebiotic, antidiabetic, antihypertensive activities and other miscellaneous bioactivities, were documented [7].

Therefore, depolymerization was recognized as the best method for producing alginate oligosaccharides. There are three popular hydrolysis methods of polysaccharides: enzymatic, physical and chemical.

Physical methods for alginate degradation are temperature, gamma, ultraviolet, ultrasound treatment or microfluidization. As a result of the interaction of physical factors on the alginate solution, the bond between the C1 and C4 atoms breaks, and thus, oligomers are formed. The use of microwaves, ionizing radiation, UV and ultrasound allows the disintegration of polysaccharides into oligomers without the interaction of strong chemical reagents. It is unnecessary to use any additives or to control environmental conditions and temperatures in the application of physical methods. The resulting depolymerization compounds are free of impurities and chemically pure [8,9].

The enzymatic hydrolysis of polysaccharides is a good choice due to the greater production of oligosaccharides. The obtained oligomers have appropriate molecular weight with the minimum amount of adverse final products as a result of chemical modification. Endo- and exo-enzymes used in that process are produced by microorganisms (bacteria and fungi). The disadvantages of this process include the possibility of microbial contamination, which needs to be removed, increasing costs. In addition, the buffer must be used, which reduces the purity of the final product. However, the efficiency and quality of the enzyme used depends on the adjustment to the environment, which consists of pH, temperature and the composition of the medium [2].

Chemical hydrolysis is a simple and cheap method, and the process is easy to conduct. The sulfuric, hydrochloric and trifluoroacetic acids and sodium periodate and hydrogen peroxide are the most common acids used in the polysaccharides’ degradation process [10,11,12]. The process normally occurs at higher temperatures above 60 °C and the reaction time varies between 2–6 h. However, acid hydrolysis may have certain disadvantages, such as the formation of toxic substances, degradation of monosaccharides and production of oligosaccharides with low efficiency. Besides, the chain length of the oligosaccharides may vary; therefore, it is necessary to monitor the reaction conditions, such as: the concentration of acid used, time, temperature, pH and conducting preliminary studies [2].

The aim of the study is to produce alginate oligomers with bioactive properties, keeping some of the functional alginate properties at the same time, by two commonly used depolymerization methods: acid and oxidation, and compare these methods. 

## 2. Materials and Methods

### 2.1. Materials 

Sodium alginate extracted from *Laminaria digitata* (particle size max. 5% > 400 μm, M:G ratio = 1.4) was supplied by Danisco, Grindsted, Denmark. 2,4,6-Tris(2-pyridyl-s-triazine) (TPTZ) was obtained from Honeywell, Seelze, Germany. 3-hydrate of sodium acetate and 2,2-diphenyl-1-Picrylhydrazyl (DPPH) were obtained from Sigma Aldrich, Poznan, Poland. Iron (III) hexahydrate and acetic acid were supplied by Chempur^®^, Piekary Slaskie Poland. Hydrogen peroxide (30%) was obtained from POCH Basic. Ethanol (96%), calcium chloride, hydrochloric acid (min. 36%) and sodium hydroxide were supplied by P.P.H. “STANLAB” Sp. J., Lublin, Poland.

### 2.2. Alginate Depolymerization

#### 2.2.1. Preparation of Alginate Oligosaccharides by Acid Hydrolysis

This method is based on the partial acid hydrolysis of sodium alginate (SA) and it was performed following the methodology described by Falkeborg et al. [13]. An aqueous solution of sodium alginate (SA) (1% *w/v*) was prepared by dissolving sodium alginate in distilled water and stirring for 24 h using the mechanical stirrer R 50 CAT (Ballrechten-Dottingen, Germany) at 400 RPM. A total of 9 mL of 3 M hydrochloric acid (HCl) was added to 300 mL of SA solution to hydrolyze heteropolymeric fractions. Such a prepared sample was heated in a water bath (Julabo TW8, Seelbach, Germany) for 20 min at 100 °C and cooled to room temperature. The cooled solution was centrifuged (Centrifuge MPW-351R, Warsaw, Poland) at 10,000 g for 20 min. The obtained precipitate containing a mixture of heteropolymeric and homopolymeric fractions was suspended in 300 mL of 0.3 M HCl and heated in a water bath for 2 h at 100 °C and cooled to room temperature. The solution was centrifuged at 10,000 g for 20 min and precipitate was neutralized with 1M NaOH and only contained the mixture of homopolymeric fractions. At this step, the sample containing mannuronic and guluronic acid (M + G) was collected. To separate mannuronate- (M) and guluronate- (G) rich fractions, the obtained solution was adjusted to pH 2.85 ± 0.05 with 1M HCl, at which polyguluronate is insoluble and polymannuronate is soluble. The sample was centrifuged at 10,000 g for 15 min and the soluble M fraction and insoluble G fraction were collected, and both were neutralized with 1M NaOH. To increase the purity of fractions M and G, they were re-adjusted to pH 2.85 ± 0.05 with 1M HCl, centrifuged and neutralized as described above. Fractions were precipitated using ethanol (ethanol:fraction ratio 2:1, *v/v*), washed with ethanol and dried at 30 °C for 20 h (Memmert 400, Büchenbach, Germany). The obtained fractions (M + G, M, G) were stored as powder.

#### 2.2.2. Preparation of Alginate Oligosaccharides by Oxidation with Hydrogen Peroxide (H_2_O_2_)

The oxidation of SA was carried out using the method described by Li et al. [14] with modifications. This method is based on the oxidation of sodium alginate by using hydrogen peroxide. An aqueous solution of sodium alginate (2% *w/v*) was prepared by dissolving SA in distilled water and stirred for 24 h on the mechanical stirrer R 50 CAT (Ballrechten-Dottingen, Germany) at 400 RPM. The oxidation process was performed on the magnetic stirrer ECM CAT (Ballrechten-Dottingen, Germany) at 600 RPM. To 150 mL of 2% AS solution, 150 mL of 10% hydrogen peroxide was added and stirred for 210 min at 25 °C. The obtained solution of hydrolysate of sodium alginate (HSA) was freeze dried using FreeZone 18L (Labconco, Kansas City, USA) and after that, stored as lyophilized powder. 

### 2.3. Hydrolysis Yield

To determine the yield of the process, the mass of used substrate and obtained hydrolysis product were weighted with an accuracy of 0.001 (Adventurer TM). The yield of the process was calculated using the equation shown below:

%Yield = m_p_/m_s_ 100 (1)

where m_p_—mass of the obtained product, m_s_—mass of the used substrate.

### 2.4. Reducing Sugar Assay and the Degree of Polymerization Calculation (DP)

The reducing sugar content was determined using 3,5-dinitrosalicylic acid (DNS) according to Sun et al. [15]. The sample was mixed with DNS reagent in a 2:1 ratio and heated for 10 min in boiling water. After cooling to room temperature, the absorbance was measured using a UV spectrophotometer UviLine 9400 (SI Analytics, Mainz, Germany) at the wavelength 540 nm. The reducing sugar content (RSC) was calculated by the following formulas:

RSC = ((A**_540_**+ 0.020)/0.554)/0.227 (2)

where A**_540_**—absorbance at 540 nm.

Then, the degree of polymerization (DP) was calculated by following equation:

DP = (RSC + 0.136)/1.085(3)

### 2.5. Fourier Transform Infrared Spectrometry

Infrared spectra were registered in a Shimadzu spectrometer (ATI Mattson, Kyoto, Japan). The transmission spectra were collected at 2 cm^−1^ resolution and by 64 scans, directly on films with a golden bridge reflexion apparatus. 

### 2.6. Rheological Characterization of Alginate Oligosaccharides 

Rheological properties were assayed using a Haake RheoStress RS6000 rotary viscometer (Thermo Scientific, Karlsruhe, Germany) following methodology described by Zimoch-Korzycka et al. [16] with modifications. The samples were 1% aqueous solutions of the tested oligosaccharides. Measurement was performed at a constant temperature (25 °C), using a cone sensor (C60/1° Ti L, diameter 20) and measuring plate (TMP60 Steel 18/8) in linear distribution CR mode. The tested samples were conditioned for 3 min at 25 °C. The shear stress was determined as a function of shear rate at the range from 0 to 100 s^−1^ following the program: 50 s to obtain maximum shear rate, 20 s at maximum shear rate, 50 s to achieve a shear rate of 0 s^−1^. Flow curves were described following Herschel–Bulkley’s and Ostwald de Waele’s rheological models. Apparent viscosity was determined at shear rate of 100 s^−1^.

The power model of Ostwald de Waele is a simple, two parameter model and it describes the flow curves of the tested medium:

τ = k × γ **^n^** (4)

or

η_α_ = k × γ ^n-1^ (5)

where τ—shear stress (Pa), k—consistency index (Pa· s), γ—shear rate (s ^−1^), n—flow behavior index (–), η_α_—apparent viscosity (mPa·s). 

The Herschel–Bulkley’s model is a simple model of flow curves for nonlinear viscoelastic fluids. Parameters describing this model are: τ_0_, k, n.

τ = τ_0_ + k × γ**^n^** (6)

where τ_0_—yield stress (Pa).

### 2.7. Antioxidant Properties of Alginate and Alginate Oligosaccharides

#### 2.7.1. Ferric Reducing Antioxidant Power (FRAP)

The ferric reducing antioxidant power was determined using the Benzie and Strain method [17]. Analysis was performed by adding 3 mL of working solution (basic solutions ratio: 1A:1B:10C) with 1 mL of 0.1% aqueous solution of the tested sample. The basic solutions were: A (1 mM TPTZ; 40 mM HCl), B (20 mM FeCl_3_ × 6H_2_O), C (0.3 mM acetate buffer pH 3.6; 3.1 g C_2_H_3_NaO_2_ × 3H_2_O and 16 mL of acetic acid made up with water to 1000 mL). After 10 min of incubation in the dark and at room temperature, absorbance at a wavelength of 593 nm was measured using a UV spectrophotometer UviLine 9400 (SI Analytics, Mainz, Germany) in plastic cuvettes. The control sample was a mixture of 3 mL of working solution and 1 mL of distilled water. The results were calculated according to calibration curve and expressed as μM Fe^2+^/mL).

#### 2.7.2. Free Radical Scavenging Activity (DPPH)

Free radical scavenging activity was determined by the method of Yen and Chen [18]. Tested samples were dissolved in water (0.1% *w/v*). The working solution was 0.3 mM ethanol solution of DPPH radicals. To determine free radical scavenging activity, 1 mL of the tested sample was mixed with 0.5 mL of freshly prepared working solution and 1 mL of ethanol. The mixture was shaken and incubated at room temperature in the dark for 30 min. Absorbance was measured in plastic cuvettes using a UV spectrophotometer UviLine 9400 (SI Analytics, Mainz, Germany) at a wavelength of 517 nm. The reference sample was 1 mL of distilled water mixed with 0.5 mL of working solution and 1 mL of ethanol. Antioxidant activity was calculated from calibration curve as the amount of Trolox needed to neutralize 0.3 mM of free DPPH radicals.

### 2.8. Antimicrobial Assay

The antimicrobial effect was tested by the broth microdilution method against *Escherichia coli* (PCM 2560) and *Staphylococcus aureus* (PCM 2602). The microorganism strains were obtained from the culture collections of the Institute of Immunology and Experimental Therapy (Polish Academy of Sciences in Wroclaw). The method was performed as described previously by Alqahtani et al. [19] with modifications. The microorganisms were grown on Mueller–Hinton Broth (MHB) (Merck, Poznań, Poland) for 18 h at 37 °C and adjusted to 1 on the McFarland standard. Then, 10 µL of the bacterial broth suspension was inoculated into each well containing 10 µL of SA, M, G, M=G and HSA in 230 µL of MHB and again incubated for 18 h at 37 °C. Bacterial growth was measured every 0.5 h using a microplate reader (Bioscreen C, Growth Curves USA, Piscataway, NJ, USA) set at an optical density (OD) of 600 nm for 48 h. 

### 2.9. Gel Preparation from Alginate Hydrolysates 

To prepare gels from obtained hydrolysates, 1% (*w/v*) aqueous solutions were prepared from each sample. The obtained solutions were stirred on the mechanical stirrer R 50 CAT (Ballrechten-Dottingen, Germany) for 60 min at 400 rpm and poured into the semi-permeable plastic cylinder-shaped casings after. To carry out gelation process casings were dipped in 0.5 M calcium chloride solution. After 24 h, obtained gels were sliced in a cylindrical shape with height of 1.5 cm and diameter of 2.5 cm. 

### 2.10. Texture Profile Analysis (TPA) of Hydrolysed Alginate Gels

Texture profile analysis was carried out on the Z010,(Zwick Roell, Ulm, Germany) texturometer for destructive deformation (75%)—gels were compressed twice with a relaxation time of 30 s. Samples were cylindrical shaped with heights of 1.5 cm and diameters of 2.5 cm and placed between parallel flat plate fixtures fitted to a TA.XT2 Texture Analyzer (Stable Micro Systems, Surrey, UK) interfaced with a computer. All samples were tested at room temperature. As a result, the following parameters were computed [20]: hardness (Hd) was defined as the maximum force during the first compression cycle (N); springiness (Sp) was the distance that the head travels during the second compression cycle (mm); cohesiveness (Coh)—the ratio of the work performed during the second compression cycle to the work recorded during the first compression of the sample (dimensionless); gumminess (Gu) was defined as the product of hardness and cohesiveness (N) and chewiness (Ch) was defined as the product of gumminess and springiness (N × mm). 

### 2.11. Statistical Data Analysis 

Statistical data analysis was performed using Statistica 12 (StatSoft, Krakow, Poland) by examining the influence of differentiating factors on the tested characteristics determined in the experiment. For this purpose, a variance analysis was used, where the differences between the meanings were determined by the Duncan test with significance level *p* ˂ 0.05.

## 3. Results and Discussion

### 3.1. Hydrolysis Yield

**Figure 1:**
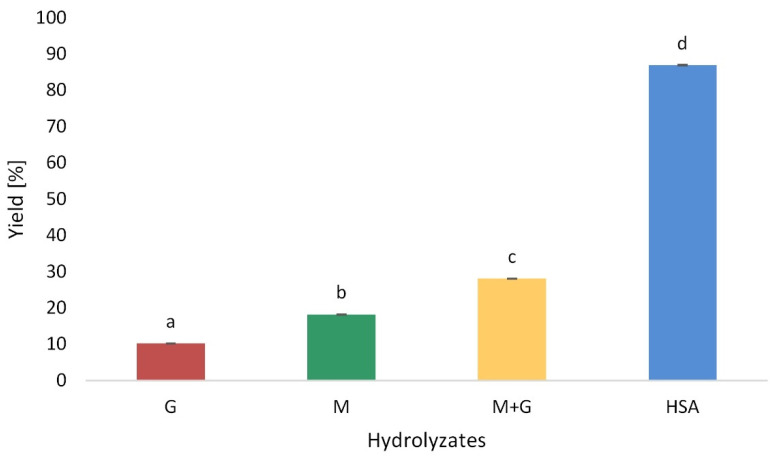
Hydrolysis yield of acidic (G, M and M + G) and oxidation (HSA) depolymerization products of sodium alginate. Results are expressed as the mean ± standard error. Values with different letters (**a–d**) within the same column differ significantly (*p* < 0.05).

The yield of the hydrolysis process is influenced by many alginate features, including composition, the sequence of individual monomers and the *M_W_* of the alginate copolymer, which varies and depends on the source or species from which the alginate is derived. Compared with the 28.1% of yield generated from the depolymerization of SA with hydrochloric acid, the yield increased to 87%, generated from the depolymerization of SA with hydrogen peroxide (Figure 1). The hydrogen peroxide caused the detachment of hydrogen atoms from the glycosidic bonds of alginate, rearranging the molecular structure and breaking glycosidic bonds [21]. The fractionation process of oligomers obtained by acidic hydrolysis resulted in obtaining yields of 10.3% and 18.2% for G and M blocks, respectively (Figure 1). Falkeborg et al. used acid hydrolysis to obtain the mannuronate- and glucuronic-rich fractions. They showed that 200–300 mg of polyglucuronate and 270–370 mg of polymannuronate can be isolated from 1000 mg of sodium alginate (Grindsted^®^ Alginate FD 170) [13]. Fenoradosoa et al., also conducted a partial hydrolysis of 1 g of sodium alginate obtained from brown algae of the *Sargassum turbinarioides* with hydrochloric acid, and obtained yields of individual fractions equal to: 30% G, 40% M and 12% M/ G [22]. The content of G blocks in commercially available formulations varies between 14.0 and 31.0% [23]. In acid hydrolysis, it is difficult to achieve 65–70% yield [24]. Hydrolysis with acid required a longer reaction time and control of the condition parameters and the yield was lower than by the oxidation method.

Mao et al. noted that the alginate depolymerization with H_2_O_2_ occurs in the first hour of reaction and the molecular weight of the depolymerized alginate decreases mainly at 20–30 ° C and further at 40 ° C. An increase in H_2_O_2_ concentration results in a gradual decrease in *M_W_*. The optimal pH range is 5–7. There are no changes when the pH rises to 8 [25]. The rate of depolymerization decreases with further pH increase [26]. Li et al. obtained depolymerized sodium alginate at 50 °C with 0.6% H_2_O_2_ after the first hour of reaction. The molecular weight of alginate decreased from 254.5 kDa to 140.9 kDa [14]. The most likely alginate degradation mechanism by oxidation is the breakage of the glycosidic bonds [25]. Very reactive free radicals are formed as the result of alginate metal ions’ decomposition of H_2_O_2_. Strong oxidizing properties of the resulting free radicals can cause the isolation of hydrogen atoms in the alginate glycosidic bonds, destroying them. It causes the formation of an aldehyde group in the new reducing end and oxidation to a carboxyl group [14].

### 3.2. Reducing Sugar Assay and the Degree of Polymerization Calculation (DP)

The results of reducing sugar content and the degree of polymerization are presented in Table 1. The significant changes after both methods of hydrolysis were noted. The native sodium alginate had the lowest RSC—2.56%—and the highest RSC was noted for the M-fraction—30.02%—which is inversely proportional to the value of DP. The linear relationship between reducing sugar concentration and DP was confirmed by Sun et al. [15]. The number of saccharide units is under 20, which confirms the obtainment of oligoalginates.

### 3.3. FTIR

**Figure 2:**
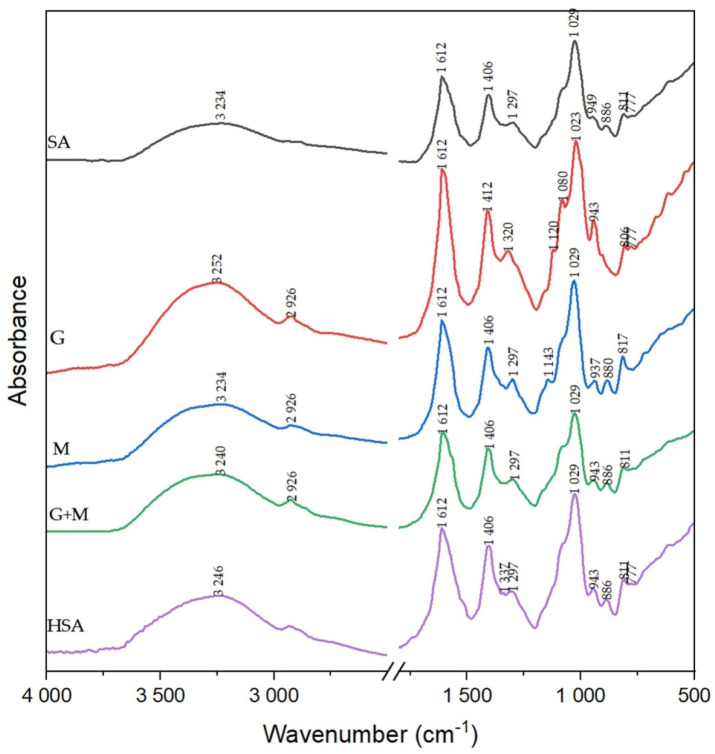
FTIR spectrum of sodium alginate (SA) and acidic (G, M and G + M fractions) and oxidation (HSA) depolymerization products.

Spectroscopic analysis was performed to confirm if alginate changed after the depolymerization processes, and the results are presented in Figure 2. The transmittance peaks of sodium alginate, which appear at 3234, 1612 and 1086 cm^−1^, are attributed to hydroxyl, C–O–O^−^, and C–O–C groups, respectively. The carboxyl groups do not change after degradation, which is why the peak at 1612 cm^−1^ for SA was taken as the reference one. Some weak bands at 1297cm^−1^ may be due to C–C–H and O–C–H deformation, and the band at 1029 cm^−1^ may be also assigned to C–O stretching vibrations. The increase in the absorption band around 2926 ± 20 and 2840 ± 20 cm^−1^ could be due to new chain ends, in correlation with the bands in the fingerprint region around 1430 and 1370 cm^−1^ [27]. The partially degraded alginates: G, M, G + M, HSA present different characteristics in the fingerprint region (1200–750 cm^−1^). The decrease in the peak ratio of the C–O–C group compared to the reference one was observed. According to Mao et al. when glycosidic bonds are breakage, the decrease in the ratio may be seen after H_2_O_2_ treatment [25]. The absorption ratios A_1086_/A_1612_ were: 0.8869, 0.8044, 0.8052, 0.8365 and 0.8576 for SA, G, M, G + M and HSA, respectively. It was proved that *M_W_* is in linear correlation with the absorption ratio A_1086_/A_1612_ [25]. The spectrum of G + M and M look similar to the SA but changed slightly in the fingerprint region. The C–O stretching vibration of the carboxylate group is moved to 937 cm^–1^ compared to G + M and SA. The spectrum of the G fraction presents the C–O stretching vibration at 943 cm^–1^. Absorption peaks at 806 and 777 cm^–1^ are attributed to deformation vibrations of COH, CCH and OCH moieties in the a-L-guluronic acid residues, with contributions of the bending deformation vibration of the C–O–C glycosidic linkage in homopolymeric blocks [28]. The HSA spectrum showed additional bands at 1337 and 777 cm^−1^, which are assigned to the skeletal vibration and deformation vibration, respectively.

### 3.4. Rheological Characterization of Alginate Oligosaccharides

**Figure 3:**
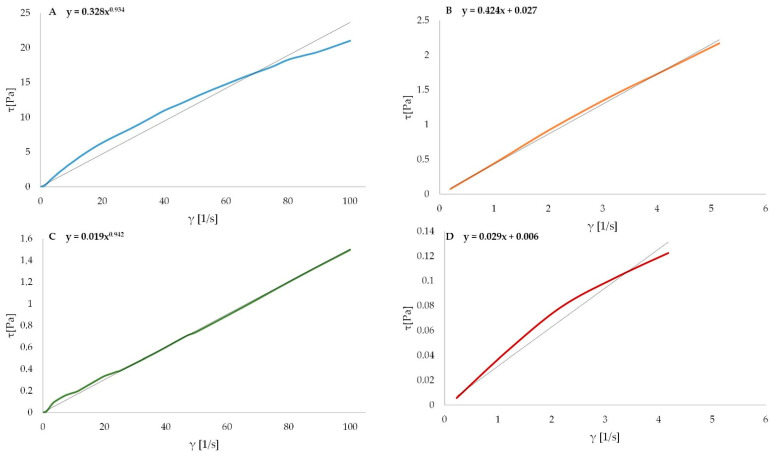
Flow curve (**A**) and yield stress determination (**B**) of SA and flow curve (**C**) and yield stress determination (**D**) of HSA.

The flow properties obtained for all the variants using the Ostwald de Waele and the Herschel-Bulkley’s models are shown in Table 2. An example of flow curves and yield stress of SA and HSA are presented in Figure 3. All tested samples presented pseudoplastic behavior, because n < 1. In consequence, each sample exhibited non-Newtonian behavior. When the molecules align at high rates of deformation, apparent viscosity is reduced and pseudoplastic behavior occurs [29]. The flow behavior index for sodium alginate solution was 0.918, and Ma obtained the n = 0.898 for a 1% solution of sodium alginate [30]. The increase in polymer concentration causes an increase in pseudoplasticity, which is reflected by a decrease in the value of the flow index n. The flow index is not sensitive to temperature changes [31]. The lowest flow behavior index was shown by the M fraction and the highest for HSA. The differences were significant statistically (*p* < 0.05), but there was no difference between SA and HSA, as well as G and M + G. Taherian et al. noticed that higher concentrations of polysaccharides cause a reduction in the flow behavior index (n) and increase in the flow consistency index (k). It means that the contact between the polymer chains in diluted solutions limits the fluid flow [32]. With an increase in the concentration of sodium alginate, the coefficient of consistency k increases [30]. The coefficient of consistency k gives an idea of the viscosity of the solution [33]. An increase in fluid viscosity causes an increase in k-value [34]. This is the reason why the statistically significant results were the same for both parameters (*p* < 0.05). There was no significant difference between all hydrolyzed samples. The different characteristics of the flow curves are dependent on the variable relationship between viscosity and shear rate [35]. The apparent viscosity decreases with increasing shear rate [30]. The apparent viscosity of hydrolyzed samples was significantly reduced in comparison to the basic sodium alginate solution. The reduction in viscosity was from the highest value, 261 mPas for SA, to the lowest value, 2 mPas for the M fraction. It was already noted, the higher *M_W_* alginate solution presents higher viscosity than lower *M_W_* at the same concentration. However, the correlation between solution viscosity and *M_W_* does not exist for microbial alginates [7]. Solutions of 0.1% sodium alginate show low viscosities at a shear rate above 10 s^−1^, indicating no intermolecular interaction due to electrostatic repulsion [36]. The decrease in viscosity may contribute to an increase in intermolecular distances due to thermal expansion with increasing temperature [37]. The structural viscosity index increases as the molecular weight of sodium alginate is increased [38]. The disruption of the sodium alginate chain may be a major factor in reducing the apparent viscosity with increasing shear rate. As Nelson et al. pointed out, the yield stress and viscous effects from a steady shear flow experiment are key properties of structured fluids [39]. The yield stress was reduced after the partial degradation of alginate and the statistical difference between samples before and after treatment was noted (*p* < 0.05). The lower viscosity of alginate may be an advantage in industrial application. Mixing high *M_W_* alginate with low *M_W_* alginates can be a way to control and obtain desire viscosity, which is easier to apply.

### 3.5. Antioxidant Properties

#### 3.5.1. Ferric Reducing Antioxidant Power (FRAP)

Previous studies have confirmed the fact that alginate has antioxidant properties, and these properties are compounded by breaking the chains of this polymer [40,41].

**Figure 4:**
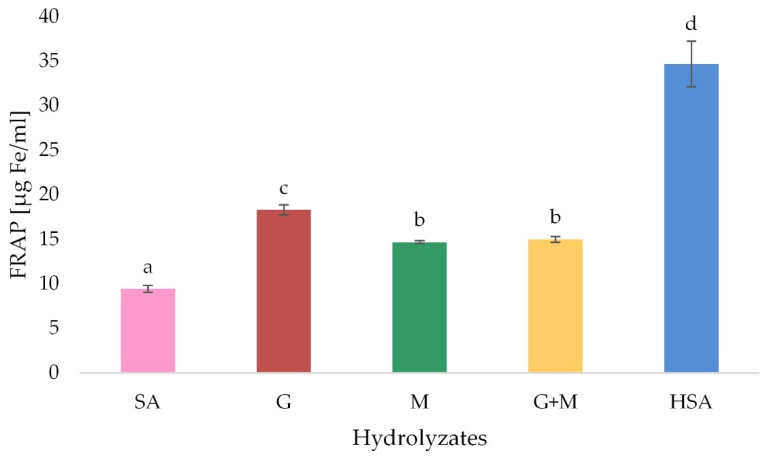
Ferric reducing antioxidant power of acidic (G, M and M + G) and oxidation (HSA) depolymerization products of sodium alginate-control (SA). Results are expressed as the mean ± standard error. Values with different letters (**a**–**d**) within the same column differ significantly (*p* < 0.05).

The highest ferric reducing antioxidant power was noted for HSA (34.7 µg), and the lowest for fractionated M blocks and G + M mixture (14.7 µg and 15.0 µg, respectively) (Figure 4). Fawzy et al. indicated that the molecular weight and M/G ratio are key factors affecting the reducing capacity of sodium alginate extracts after alkali treatment. The alkaline pH may enhance the antioxidant properties of alginate [42]. in the lowest *M_W_* and G/M ratio, the highest radical scavenging activity can be obtained [41]. Additionally, Kelishomi et al. confirmed the higher antioxidant potential of alginate upon a decrease in molecular weight [43]. In our study, the hydrolysis significantly improved the ferric reducing antioxidant power of tested samples compared to native sodium alginate. The degree of polymerization was 3.60, 4.19, 5.14 and 5.68 for M, G, M + G and HSA, respectively, which suggest their lower molecular weight which is in agreement with Kelishomi et al. [43]. The ferric reducing antioxidant power of the sodium alginate derivatives depends as well on temperature and pH. However, Falkeborg et al. noted that the alginate oligosaccharides (AOs) prepared by alginate lyase present the insignificant ferrous ion chelating activity of AOs, which is insignificant from a food preservation point of view [13].

#### 3.5.2. Free Radical Scavenging Activity (DPPH)

**Figure 5:**
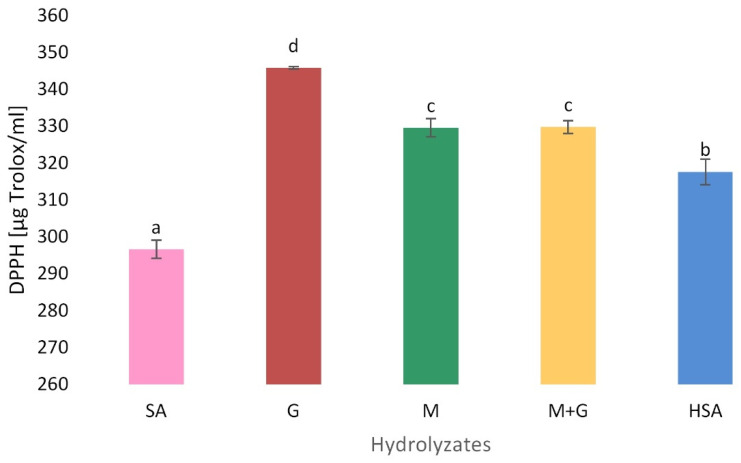
Free radical scavenging activity of acidic (G, M and M + G) and oxidation (HSA) depolymerization products of sodium alginate (SA). Results are expressed as the mean ± standard error. Values with different letters (a–d) within the same column differ significantly (*p* < 0.05).

DPPH free radicals have a stable violet color in polar solutions, while in contact with the antioxidant, they discolor after the capture of the hydrogen atom, becoming non-radical. Figure 5 shows the free radical scavenging activity of origin sodium alginate, acidic fractionated (G, M and M + G fractions) and depolymerized by oxidation (HSA). The G fraction showed the highest hydroxyl radical scavenging activity (346 µg/Trolox ml). The M fraction (330 µg/Trolox ml), the mixture of M+G (330 µg/Trolox ml), as well as hydrolysates obtained after alginate oxidation (318 µg/Trolox ml) had significantly lower antioxidant potential than the G fraction. (*p* < 0.05). The lowest radical scavenging effect on the DPPH free radical was presented by origin SA (297 µg/Trolox ml). The results are in accordance with Sari-Chmayssem et al., who observed the higher hydroxyl radical scavenging activity for the PolyG fractions in the highest tested concentration (~92%) and this activity was comparable with those of standard antioxidants. They concluded that rotation around the glycosidic linkage, which is responsible for G blocks’ flexibility, was hindered by α (1–4) glycosidic linkage in the G blocks, causing their stiffness and influencing the availability of sodium alginate hydroxyl groups [44]. The possible antioxidant mechanism of sodium alginate proposed in the literature is the result of the hydrogen atom transfer and double bond formation by depolymerization between C-4 and C-5 in time [43]. Sen confirmed that the radical scavenging efficacy is related to the G content. The lower the G-block content, the lower the antioxidant capacity [45].

### 3.6. Antimicrobial Activity

**Figure 6:**
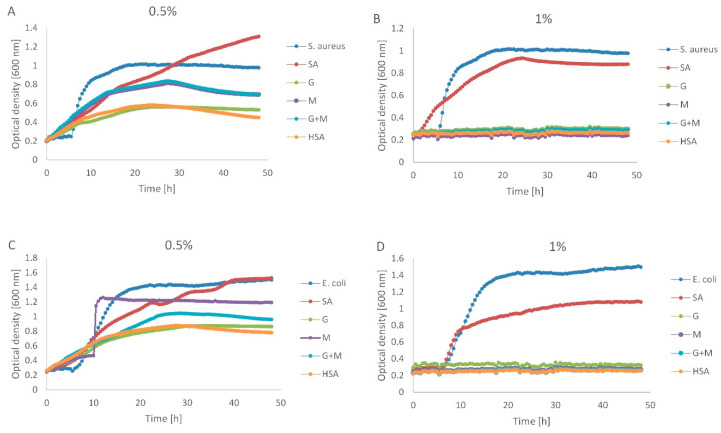
Growth curves of Staphylococcus aureus in the presence and absence of acidic (G, M and M + G) and oxidation (HSA) depolymerization products of sodium alginate (SA) in the concentration of 0.5% (**A**) and 1% (**B**). Growth curves of Escherichia coli in the presence and absence of acidic (G, M and M + G) and oxidation (HSA) depolymerization products of sodium alginate (SA) in the concentration of 0.5% (**C**) and 1% (**D**). Points are means of three measurements.

The antimicrobial activities of native (SA) and depolymerized alginate (G, M, M + G and HSA) in liquid medium were assessed on Staphylococcus aureus and Escherichia coli strains. Figure 6 shows the growth curves of (A and B) S. aureus and (C and D) E. coli in the absence and presence of SA, G, M, G + M and HSA in the concentration of 0.5% and 1%. Native (SA) and depolymerized alginate (G, M, M + G and HSA) at a concentration of 0.5% did not inhibit the growth of Staphylococcus aureus and Escherichia coli. Under these conditions, alginate oligomers merely limited the growth of the tested strains (expressed as optical density). The ΔOD ion these conditions was in the range of 0.2–0.8, while in the control cultures, this parameter was 1.2–1.3. The complete growth inhibition (OD = 0) was, however, observed in the presence of 1% native, as well as depolymerized alginate. Hu et al. tested the antibacterial activity of depolymerized alginate and found out the M fractions showed higher antibacterial activities than the G fractions. Smaller molecules of the M fraction are easier to pass through bacterial envelopes to obtain the antimicrobial effect [46]. Simultaneously, the antimicrobial effects of the alginate oligomer OligoG CF-5/20 against Gram-negative bacteria was confirmed by Pritchard [47].

### 3.7. TPA

The texture profile analysis imitates the action of the jaw, and based on the force–time curve obtained after analysis, several textural parameters, which correspond with sensory evaluation [20]. The alginate molecule is a linear copolymer, composed of two types of monomers: α-L-guluronic acid (G) and β-Dmannuronic acid (M). Monomer molecules are epimers and have reverse spatial arrangement in the position of carbon C5. In the presence of divalent metal ions, such as Ba^2+^, Ca^2+^, Mg^2+^, Sr^2+^, a strong interaction with the COO^–^ groups of guluronic acid of alginate chains is formed, resulting in a gel structure called “egg box”. The participation of individual blocks and their distribution in alginates determine the textural properties of the gels made of them. Rigid and brittle gels are made of alginate with a high content of G-blocks, while alginates with a high content of M blocks make it possible to obtain gels that are weak, elastic, and prone to deformation, but retain water. As with other polysaccharides, differences in the properties of alginates also depend on their molecular weight and degree of polymerization [48]. It is well known that the gel-forming ability of alginates depends on the presence of homopolyguluronic acid chains. This is the reason why only AS and ASH were capable of forming a gel structure and were subjected to examination of the texture profile (Table 3). Despite the addition of calcium chloride, the M and G fractions did not develop a stable gel structure. In the G fraction, only local agglomerates were observed, but were not stable. The M and G fractions, as well as M + G, may be used as enrichment preparates with strong antioxidant and antibacterial properties even if they lost their gelling ability. The significant differences between SA and HSA were noted for each tested textural parameter: hardness, springiness, cohesiveness, gumminess and chewiness (*p* < 0.05). The hardness of the SA gel became lower after H_2_O_2_ treatment, from 71.25 to 13.68 N. The cohesiveness of the formed gels was between 0.16 and 0.40 for HSA and SA, respectively. The low value of the cohesiveness means that obtained gels are more plastic than elastic, which is a desirable feature in designing easy-to-chew food. The springiness or elasticity of tested gels lowered after controlled depolymerization from 0.46 to 0.42 mm. This means that both gels possess a low elastic property, which was also confirmed by the result of springiness. When the gumminess of the gel is higher, the hardness becomes higher too. It was found that when the gumminess and chewiness of the gel are lowered, the hardness of the gel is also decreased. A similar effect was noted by other authors [49,50]. Larsen et al. studied the influence of the molecular weight and alginate concentration on the gel formation and noted that degraded alginate by autoclaving from 257 kDa to 70 kDa caused a higher gelling rate. Authors explained *M_W_* dependency as the result of the faster availability of shorter chains during gelling [51]. Alginate also formed gels in low pH of 2.8–4.0, but they are weaker than ionic gels [52]. Park et al. obtained a gel-like matrix with alginate oligosaccharides to protect the lysosome at low pH. However, when the pH rose, the alginate oligosaccharides dissolved and released the enzyme [53].

## 4. Conclusions

The hydrogen peroxide method is easier to prepare due to the absence of acids or bases, and the reaction time is much shorter—less than 4 h. Compared to acid hydrolysis, the HSA was obtained with much higher yield after using H_2_O_2_ degradation. Higher efficiency may be caused because there is no need to centrifuge, filter and transfer the solutions several times, which leads to large losses of products during the acid hydrolysis process. This suggests that the 5% H_2_O_2_ oxidation method is better for potential industrial use. Controlled radical hydrolysis is also a green chemistry process, which is an additional advantage. The alginate oligosaccharides may be used as an additive to the alginate with high *M_W_* as a way of lowering the viscose and enriching final products with antioxidant and antimicrobial properties; for example, to stabilize and extend the shelf life of mayonnaise or Greek yoghurt.

## Figures and Tables

**Table 1:** Reducing sugar content and the calculated degree of depolymerization of native and hydrolysated alginate.

Parameters	Variants				
	SA	G	M	M+G	HSA
RSC (%)	2.56 ^a^ ± 0.02	25.74 ^d^ ± 0.06	30.02 ^e^ ± 0.08	20.97 ^c^ ± 0.08	18.95 ^b^ ± 0.06
DP (–)	40.28 ^e^ ± 0.34	4.19 ^b^ ± 0.01	3.60 ^a^ ± 0.01	5.14 ^c^ ± 0.02	5.68 ^d^ ± 0.02

^a–e^ values with different letters within the same column differ significantly (*p* < 0.05).

**Table 2:** Flow properties.

Variants	Ostwald de Waele Model		Herschel–Bulkley’s Model	Apparent Viscosity η (mPa•s)
	Consistency Index k (Pa·s)	Flow Behavior Index n (-)	Yield Stress τ_0_ (Pa)	
SA	0.334 ^a^ ± 0.017	0.918 ^a^ ± 0.066	0.030 ^a^ ± 0.003	261.140 ^a^ ± 6.025
G	0.009 ^b^ ± 0.003	0.869 ^ab^ ± 0.123	0.005 ^b^ ± 0.002	3.580 ^b^ ± 1.032
M	0.003 ^b^ ± 0.002	0.742 ^b^ ± 0.050	0.002 ^b^ ± 0.001	2.330 ^b^ ± 0.250
M+G	0.004 ^b^ ± 0.002	0.822 ^ab^ ± 0.039	0.003 ^b^ ± 0.002	4.130 ^b^ ± 0.191
HSA	0.017 ^b^ ± 0.002	0.955 ^a^ ± 0.025	0.006 ^b^ ± 0.002	15.010 ^b^ ± 0.302

^a–b^ values with different letters within the same column differ significantly (*p* < 0.05).

**Table 3:** Texture profile analysis (TPA) parameters of sodium alginate and alginate.

Variants	Hd (N)	Sp (mm)	Coh (–)	Gu (N)	Ch (Nxmm)
SA	71.25 ^a^ ± 3.99	0.46 ^a^ ± 0.01	0.40 ^a^ ± 0.02	28.50 ^a^ ± 2.46	13.11 ^a^ ± 1.23
HSA	13.68 ^b^ ± 1.81	0.42 ^b^ ± 0.02	0.16 ^b^ ± 001	2.19 ^b^ ± 0.39	0.92 ^b^ ± 0.20

^a–b^ values with different letters within the same column differ significantly (*p* < 0.05).

## Data Availability

The data presented in this study are available on request from the corresponding author.

## References

[1] Liu J., Willför S., Xu C. (2015). A review of bioactive plant polysaccharides: Biological activities, functionalization, and biomedical applications. Bioact. Carbohydr. Diet. Fibre..

[2] De Moura F.A., Macagnan F.T., Da Silva L.P. (2015). Oligosaccharide production by hydrolysis of polysaccharides: A review. Int. J. Food Sci. Technol..

[3] Patel S., Goyal A. (2011). Functional oligosaccharides: Production, properties and applications. World J. Microbiol. Biotechnol..

[4] Abd El-Mohdy H.L. (2019). Radiation-induced degradation of sodium alginate and its plant growth promotion effect. Arab. J. Chem..

[5] Piacentini E., Drioli E., Giorno L. (2016). Encapsulation Efficiency. Encyclopedia of Membranes.

[6] Labre F., Mathieu S., Chaud P., Morvan P.Y., Vallée R., Helbert W., Fort S. (2018). DMTMM-mediated amidation of alginate oligosaccharides aimed at modulating their interaction with proteins. Carbohydr. Polym..

[7] Liu J., Yang S., Li X., Yan Q., Reaney M.J.T., Jiang Z. (2019). Alginate Oligosaccharides: Production, Biological Activities, and Potential Applications. Compr. Rev. Food Sci..

[8] Muley A.B., Ladole M.R., Suprasanna P., Dalvi S.G. (2019). Intensification in biological properties of chitosan after γ-irradiation. Int. J. Biol. Macromol..

[9] Wei Y., Wang C., Liu X., Mackie A., Zhang L., Liu J., Mao L., Yuan F., Gao Y. (2020). Impact of microfluidization and thermal treatment on the structure, stability and in vitro digestion of curcumin loaded zein-propylene glycol alginate complex nanoparticles. Food Res. Int..

[10] Khajouei A.R., Keramat J., Hamdami N., Ursu A.V., Delattre C., Laroche C., Gardarin C., Lecerf D., Desbrières J., Djelveh G. (2018). Extraction and characterization of an alginate from the Iranian brown seaweed Nizimuddinia zanardini. Int. J. Biol. Macromol..

[11] Huamani-Palomino R.G., Jacinto C.R., Alarcón H., Mejía I.M., López R.C., de Oliveira Silva D., Cavalheiro E.T.G., Venâncio T., Dávalos J.Z., Valderrama A.C. (2019). Chemical modification of alginate with cysteine and its application for the removal of Pb(II) from aqueous solutions. Int. J. Biol. Macromol..

[12] Zhu Q., Wu S. (2019). Water-soluble β-1,3-glucan prepared by degradation of curdlan with hydrogen peroxide. Food Chem..

[13] Falkeborg M., Cheong L.Z., Gianfico C., Sztukiel K.M., Kristensen K., Glasius M., Xu X., Guo Z. (2014). Alginate oligosaccharides: Enzymatic preparation and antioxidant property evaluation. Food Chem..

[14] Li X., Xu A., Xie H., Yu W., Xie W., Ma X. (2010). Preparation of low molecular weight alginate by hydrogen peroxide depolymerization for tissue engineering. Carbohydr. Polym..

[15] Sun M., Sun C., Xie H., Yan S., Yin H. (2019). A simple method to calculate the degree of polymerization of alginate oligosaccharides and low molecular weight alginates. Carbohydr Res..

[16] Zimoch-Korzycka A., Kulig D., Jarmoluk A., Marycz K., Matuszczak W. (2016). Study of Enzymatically Treated Alginate/Chitosan Hydrosols in Sponges Formation Process. Polymers.

[17] Benzie I.F.F., Strain J.J. (1996). The ferric reducing ability of plasma (FRAP) as a measure of “Antioxidant Power”: The FRAP assay. Analytic. Biochem..

[18] Yen G.C., Chen H.Y. (1995). Antioxidant activity of various tea extracts in relation to their antimutagenicity. J. Agric. Food Chem..

[19] Alqahtani F.Y., Aleanizy F.S., El Tahir E., Alquadeib B.T., Alsarra I.A., Alanazi J.S., Abdelhady H.G. (2019). Preparation, characterization, and antibacterial activity of diclofenac-loaded chitosan nanoparticles. Saudi Pharm. J..

[20] Bourne M.C. (2002). Food Texture and Viscosity: Concept and Measurement.

[21] Yang Z., Li J.P., Guan H.S. (2010). Preparation and characterization of oligomannuronates from alginate degraded by hydrogen peroxide. Carbohydr. Polym..

[22] Fenoradosoa T.A., Ali G., Delattre C., Laroche C., Petit E., Wadouachi A., Michaud P. (2010). Extraction and characterization of an alginate from the brown seaweed Sargassum turbinarioides Grunow. J. Appl. Phycol..

[23] Qin Y. (2008). Alginate fibres: An overview of the production processes and applications in wound management. Polym. Int..

[24] Du B., Song Y., Hu X., Liao X., Ni Y., Li Q. (2011). Oligosaccharides prepared by acid hydrolysis of polysaccharides from pumpkin (Cucurbita moschata) pulp and their prebiotic activities. Int. J. Food Sci. Technol..

[25] Mao S., Zhang T., Sun W., Ren X. (2012). The depolymerization of sodium alginate by oxidative degradation. Pharm. Develop. Technol..

[26] Martinez J.M.L., Denis M.F.L., Piehl L.L., de Celis E.R., Buldain G.Y., Orto V.C.D. (2008). Studies on the activation of hydrogen peroxide for color removal in the presence of a new Cu(II)-polyampholyte heterogeneous catalyst. Appl. Catal. B.–Environ..

[27] Dima S.-O., Panaitescu D.-M., Orban C., Ghiurea M., Doncea S.-M., Fierascu R.C., Nistor C.L., Alexandrescu E., Nicolae C.-A., Trică B. (2017). Bacterial Nanocellulose from Side-Streams of Kombucha Beverages Production: Preparation and Physical-Chemical Properties. Polymers.

[28] Leal D., Matsuhiro B., Rossi M., Caruso F. (2008). FT-IR spectra of alginic acid block fractions in three species of brown seaweeds. Carbohydr Res..

[29] Batalha L.S., Pardini Gontijo M.T., Novaes de Carvalho Teixeira A.V., Gouvêa Boggione D.M., Soto Lopez M.E., Eller M.R., Santos Mendonça R.C. (2021). Encapsulation in alginate-polymers improves stability and allows controlled release of the UFV-AREG1 bacteriophage. Int. Food Res. J..

[30] Ma J., Lin Y., Chen X., Zhao B., Zhang J. (2014). Flow behavior, thixotropy and dynamical viscoelasticity of sodium alginate aqueous solutions. Food Hydrocoll..

[31] Moraes I.C.F., Fasolin L.H., Cunha R.L., Menegalli F.C. (2011). Dynamic and steady-shear rheological properties of xanthan and guar gums dispersed in yellow passion fruit pulp (Passiflora edulis f. flavicarpa). Braz. J. Chem. Eng..

[32] Taherian A.L., Lacasse P., Bisakowski B., Pelletier M., Lanctôt A., Fustier P. (2017). Rheological and thermogelling properties of commercials chitosan/β-glycerophosphate: Retention of hydrogel in water, milk and UF-milk. Food Hydrocoll..

[33] Björn A., Segura de La Monja P., Karlsson A., Ejlertsson J., Svensson B.H., Kumar S. (2012). Rheological Characterization.

[34] Gómez-Díaz D., Navaza J.M. (2003). Rheology of aqueous solutions of food additives: Effect of concentration, temperature and blending. J. Food Eng..

[35] Keleşoğlu S., Pettersen B.H., Sjöblom J. (2012). Flow properties of water-in- North Sea heavy crude oil emulsions. J. Pet. Sci. Eng..

[36] Yang J., Chen S., Fang Y. (2009). Viscosity study of interactions between sodium alginate and CTAB in dilute solutions at different pH values. Carboh. Polym..

[37] Xiao Q., Tong Q., Lim L.-T. (2012). Pullulan-sodium alginate based edible films: Rheological properties of film forming solutions. Carboh. Polym..

[38] Guo X., Zhu P., Wang B., Zhan Y., Zhang J. (2007). Study on rheological behavior of sodium alginate and its blend solution. China Synt. Fib. Ind..

[39] Nelson A.Z., Schweizer K.S., Rauzan B.M., Nuzzo R.G., Vermant J., Ewoldt R.H. (2019). Designing and transforming yield-stress fluids. Curr. Opin. Solid State Mater. Sci..

[40] Zhao X., Li B., Xue C., Sun L. (2012). Effect of molecular weight on the antioxidant property of low molecular weight alginate from Laminaria japonica. J. Appl. Phycol..

[41] Şen M., Atik H. (2012). The antioxidant properties of oligo sodium alginates prepared by radiation-induced degradation in aqueous and hydrogen peroxide solutions. Radiat. Phys. Chem..

[42] Fawzy M.A., Goma A.M., Hifney A.F., Abdel-Gawad K.M. (2017). Optimization of alginate alkaline extraction technology from Sargassum latifolium and its potential antioxidant and emulsifying properties. Carboh. Polym..

[43] Kelishomi Z.H., Goliaei B., Mahdavi H., Nikoofar A., Rahimi M., Moosavi-Movahedi A.A., Bigdeli B. (2016). Antioxidant activity of low molecular weight alginate produced by thermal treatment. Food Chem..

[44] Sari-Chmayssem N., Taha S., Mawlawi H. (2016). Extracted and depolymerized alginates from brown algae Sargassum vulgare of Lebanese origin: Chemical, rheological, and antioxidant properties. J Appl Phycol..

[45] Sen M. (2011). Effects of molecular weight and ratio of guluronic acid to mannuronic acid on the antioxidant properties of sodium alginate fractions prepared by radiation-induced degradation. Appl. Radiat. Isot..

[46] Hu X., Jiang X., Gong J. (2005). Antibacterial activity of lyase-depolymerized products of alginate. J. Appl. Phycol..

[47] Pritchard M., Powell L., Khan S. (2017). The antimicrobial effects of the alginate oligomer OligoG CF-5/20 are independent of direct bacterial cell membrane disruption. Sci. Rep..

[48] Rhein-Knudsen N., Tutor Ale M., Ajalloueian F., Meyer A.S. (2017). Characterization of alginates from Ghanaian brown seaweeds: Sargassum spp. and Padina spp.. Food Hydrocolloids.

[49] Mutlu C., Tontul S.A., Erbaş M. (2018). Production of a minimally processed jelly candy for children using honey instead of sugar. LWT.

[50] Yusof N., Jaswir I., Jamal P., Jami M. (2019). Texture Profile Analysis (TPA) of the jelly dessert prepared from halal gelatin extracted using High Pressure Processing (HPP). Mal. J. Fund. Appl. Sci..

[51] Larsen B.E., Bjørnstad J., Pettersen E.O., Tønnesen H.H., Melvik J.E. (2015). Rheological characterization of an injectable alginate gel system. BMC Biotechnol..

[52] Roopa B.S., Bhattacharya S. (2008). Alginate gels: I. Characterization of textural attributes. J. Food Eng..

[53] Park R.M., Nguyen N.H.T., Lee S.M., Kim Y.H., Min J. (2021). Alginate oligosaccharides can maintain activities of lysosomes under low pH condition. Sci Rep..

